# Nurse-Led Binaural Beat Intervention for Anxiety Reduction in Pterygium Surgery: A Randomized Controlled Trial

**DOI:** 10.3390/nursrep15080282

**Published:** 2025-07-31

**Authors:** Punchiga Ratanalerdnawee, Mart Maiprasert, Jakkrit Klaphajone, Pongsiri Khunngam, Phawit Norchai

**Affiliations:** 1 Department of Anti-Aging and Regenerative Medicine, College of Integrative Medicine, Dhurakij Pundit University, Bangkok 10210, Thailand; mart.mai@dpu.ac.th (M.M.); pongsiri.koo@dpu.ac.th (P.K.); phawit.nor@dpu.ac.th (P.N.); 2 Department of Rehabilitation Medicine, Faculty of Medicine, Chiang Mai University, Chiang Mai 50200, Thailand; jakkrit.k@cmu.ac.th

**Keywords:** nurse-led intervention, binaural beats, perioperative anxiety, music therapy, patient-centered care, pterygium surgery, local anesthesia

## Abstract

**Background/Objectives**: Anxiety before ophthalmic surgery under local anesthesia may hinder patient cooperation and surgical outcomes. Nurse-led auditory interventions offer a promising non-pharmacological approach to perioperative anxiety management. This study evaluated the effectiveness of superimposed binaural beats (SBBs)—classical music layered with frequency differentials—in reducing anxiety during pterygium surgery with conjunctival autografting. **Methods**: In this randomized controlled trial, 111 adult patients scheduled for elective pterygium excision with conjunctival autografting under local anesthesia were allocated to one of three groups: SBBs, plain music (PM), or silence (control). A trained perioperative nurse administered all auditory interventions. The patients’ anxiety was assessed using the State–Trait Anxiety Inventory—State (STAI-S), and physiological parameters (blood pressure, heart rate, respiratory rate, and oxygen saturation) were recorded before and after surgery. **Results**: The SBB group showed significantly greater reductions in their STAI-S scores (*p* < 0.001), systolic blood pressure (*p* = 0.011), heart rate (*p* = 0.003), and respiratory rate (*p* = 0.009) compared to the PM and control groups. No adverse events occurred. **Conclusions:** SBBs are a safe, nurse-delivered auditory intervention that significantly reduces perioperative anxiety and supports physiological stability. Their integration into routine nursing care for minor ophthalmic surgeries is both feasible and beneficial. Trial Registration: This study was registered with the Thai Clinical Trials Registry (TCTR) under registration number TCTR20250125002 on 25 January 2025.

## 1. Introduction

Pterygium is a common eye condition in which a triangular tissue grows from the conjunctiva—the thin membrane covering the white part of the eye—onto the cornea, usually starting from the inner (nasal) side. This growth often causes symptoms such as eye redness, irritation, dryness, or a sensation of having something in the eye. In more advanced cases, it may extend toward the center of the cornea, potentially affecting vision by altering the eye’s surface or blocking the line of sight [1].

Globally, pterygium is considered a prevalent ocular surface disorder, with a pooled prevalence of approximately 10.2%. Higher rates are observed in people living in low-latitude (sun-intensive) regions and among older adults. The prevalence increases with age—rising from 11% in individuals aged 40–49 years to over 20% in those aged 60 and above—and is more common among males and those living in rural areas, likely due to greater exposure to sunlight and outdoor environments [2]. The pathogenesis of pterygium is multifactorial, involving chronic ultraviolet (UV) radiation, viral infections, and the heightened expression of inflammatory mediators and growth factors. Additional mechanisms—including oxidative stress, tumor suppressor gene dysfunction, apoptosis, and neuropeptide activity—have been implicated in the abnormal proliferation of conjunctival tissue across the cornea [3].

Surgical excision with conjunctival autografting is widely regarded as the current standard treatment for clinically significant or progressive pterygium, owing to its lower recurrence rate compared to other techniques such as bare sclera excision [4]. In ophthalmic surgery, nurses play a central role in patient preparation and the provision of psychological support, ensuring emotional readiness and procedural cooperation. This includes efforts to reduce preoperative anxiety through patient-centered communication, as supported by evidence from randomized controlled trials demonstrating that a standardized preoperative nurse–patient dialog can significantly alleviate anxiety levels prior to surgery [5].

Despite the minimally invasive nature of the procedure, ophthalmic surgeries often provoke considerable anxiety, stemming from fears of vision loss, unfamiliar surgical settings, or concerns about intraoperative discomfort and postoperative recovery [6]. Such psychological distress can activate the sympathetic nervous system, leading to elevations in blood pressure, heart rate, and respiratory rate—physiological responses that may compromise patient cooperation and the surgical outcome [7].

Managing perioperative anxiety is therefore a critical aspect of comprehensive patient care. In ambulatory ophthalmic surgery settings, where pharmacologic sedation is often limited, nurse-led interventions may play a supportive role in mitigating psychological distress. Nurses contribute significantly to perioperative emotional stabilization through environmental control, psychological reassurance, and the implementation of evidence-based, non-pharmacological strategies [8].

Auditory stimulation—particularly using binaural beats within the alpha frequency range (8–13 Hz)—has demonstrated efficacy in reducing anxiety by facilitating cortical relaxation, as evidenced by increased alpha wave activity and decreased anxiety scores in randomized clinical studies [9]. A growing body of evidence supports the clinical application of such interventions across diverse perioperative contexts [10,11,12]. The integration of binaural beats with music may produce synergistic anxiolytic effects, as observed in ophthalmic procedures such as cataract surgery [13].

Superimposed binaural beats (SBBs) represent an emerging auditory intervention designed to transition brainwave activity toward relaxed states more efficiently than conventional binaural beats. Unlike traditional binaural beats—which are typically produced by differentiating two pure-tone sine wave frequencies—SBBs incorporate additional layers of stimulation. In this study, supplementary beats were synthesized from the frequency-shifted sound waves of classical musical instruments, creating a richer and more dynamic auditory experience [14]. Despite their promising neurophysiological mechanisms, the clinical application of SBBs as a nurse-delivered modality remains underexplored, particularly in ophthalmic surgical populations.

The aim of this study was to evaluate the anxiolytic efficacy of SBBs among patients undergoing pterygium surgery under local anesthesia. Using a randomized controlled design, we investigated whether a structured, nurse-led auditory intervention could reduce perioperative anxiety and stabilize patients’ physiological responses. The results may support the use of sensory-based, non-pharmacological strategies in nursing care and contribute to the evolving role of nurses in providing patient-centered interventions in outpatient surgical settings.

## 2. Materials and Methods

### 2.1. Study Design and Participants

This study employed a parallel-group randomized controlled trial (RCT) design with a double-blinded superiority framework to evaluate the efficacy of SBBs in reducing perioperative anxiety. The trial was approved by the Dhurakij Pundit University Human Research Ethics Committee (Approval Code: COA No. 007/67) and conducted in full accordance with the approved protocol. The trial was prospectively registered in the Thai Clinical Trials Registry (TCTR) under the identifier TCTR20250125002 on 25 January 2025.

Participant recruitment was conducted between January and May 2025 at Phra Ajan Baen Thanagro Hospital, Thailand. Eligible individuals were adults aged 30 years or older scheduled for elective pterygium excision with conjunctival autografting under local anesthesia—a surgical technique requiring extended operative time and increased patient cooperation due to autologous tissue harvesting and graft placement. Exclusion criteria included complex pterygium morphology, auditory impairment, active ear infections, a prior history of epilepsy, cognitive dysfunction, psychiatric disorders, a prior history of substance use, or preoperative blood pressure exceeding 160/100 mmHg.

Participants were enrolled using consecutive sampling based on their scheduled surgery appointments and fulfillment of eligibility criteria. All were invited verbally by outpatient department (OPD) nurses and participated voluntarily. All patients had been pre-scheduled for pterygium excision with conjunctival autografting under local anesthesia as part of routine clinical care.

On the day of surgery, following clinical evaluation—which included medical history, physical examination, audiologic and ophthalmologic assessments—informed consent was obtained by trained OPD nurses at the outpatient clinic at least 60 min prior to surgery during the preoperative preparation phase. At this time, the participants received both verbal and written information regarding the study’s objectives, procedures, potential risks, and their right to withdraw at any point without affecting their care.

To ensure data confidentiality and participant privacy, all collected data were anonymized using unique study codes. Only the principal investigator, who also served as the designated data manager, had access to the password-protected files. All data will be securely stored on an encrypted institutional server for a period of five years following the completion of the study, after which it will be permanently deleted.

The participants were randomly allocated to three groups using a computer-generated randomization scheme: (1) the SBB group, who received classical music embedded with superimposed binaural frequencies; (2) the plain music (PM) group, who received the same music without binaural components; and (3) the control group, who wore headphones without any audio input.

The operating ophthalmologist, the four rotating perioperative nurses responsible for questionnaire administration and vital sign monitoring, and all the outcome assessors were blinded to the group allocation. Although the intervention nurse could not be blinded due to the nature of the auditory protocol, all the participants remained masked to their assigned group in order to minimize expectation bias.

No interim analyses or stopping rules were implemented. The study proceeded without any deviation from the registered protocol. The potential adverse effects—such as dizziness, headaches, nausea, or discomfort from headphone use—were pre-specified and continuously monitored throughout the perioperative period. Monitoring was embedded into routine nursing surveillance procedures throughout the intervention period.

### 2.2. Interventions

Prior to enrollment, all participants underwent audiometric screening using the MAICO Audiometer MA 25 (MAICO Diagnostics GmbH, Berlin, Germany) to confirm normal hearing function. All the individuals passed the screening with auditory thresholds ≤ 25 dB HL at 500–2000 Hz, ensuring the accurate perception of auditory stimuli.

SBB Group: The participants assigned to the SBB group received a custom-designed auditory track developed and licensed by Chiang Mai University, Thailand. The audio incorporated ambient natural sounds—such as water streams, forest ambiance, and ocean waves—combined with superimposed binaural layers. These additional layers were synthesized by frequency-shifting the sound waves of individual musical instruments to supplement the primary binaural beat, which was generated via pure-tone sine wave differentiation. The SHARM self-hypnosis system (Version 2.4; Cyber Team Ltd. and Informer Technologies Inc., Madrid, Spain) was employed, and the auditory sequence began at 20 Hz (beta range) and remained there for the first 5 min, gradually decreased to 10 Hz (alpha range) over the next 5 min, and then remained at 10 Hz for the remainder of the session. The participants listened to the audio throughout the surgical procedure and continued wearing the headphones for up to 15 min postoperatively. Although the full 60 min track was not always completed, auditory exposure remained consistent across the participants. All the files were encoded in an MP3 format, with the binaural layering rendered imperceptible to conscious awareness.

PM Group: The participants in the plain music (PM) group listened to the same musical composition used in the SBB group but without the embedded binaural frequencies. The musical and ambient elements were preserved, while the frequency differentials necessary for binaural beat generation were intentionally excluded. The audio parameters—such as the volume, duration, and structural progression—were matched to those of the SBB track to ensure comparability between the groups.

Control Group: The participants assigned to the control group wore identical headphones but did not receive any auditory input, serving as a sham intervention. This setup controlled for environmental and equipment-related variables without introducing auditory stimulation.

Delivery Method and Timing: All the participants used the same standardized audio equipment, comprising a Sony NW-A306 A300 Walkman^®^ (Sony Corporation, Tokyo, Japan) connected via Bluetooth to Bose QuietComfort^®^ 45 noise-canceling headphones (Bose Corporation, Framingham, MA, USA). In both the SBB and PM groups, the auditory interventions were initiated 10 min prior to the surgical procedure, continued throughout the operation, and extended for an additional 15 min postoperatively. The audio volume was initially preset at a soft level—approximately 10 out of 30 on the Walkman^®^ interface—corresponding to a sound level comparable to that of soft conversational speech. This level ensured auditory perception while minimizing the risk of discomfort or hearing fatigue. Before initiating playback, the responsible nurse performed an informal sound check to confirm that each participant could perceive the audio comfortably. Minor individual adjustments were permitted to accommodate personal listening preferences. During the procedure, the participants were also allowed to fine-tune the volume within a comfortable range. The headphones were set to “Aware Mode” to ensure participants remained responsive to intraoperative verbal instructions, thereby maintaining communication and patient safety.

Anesthesia Protocol: Topical anesthesia was applied using 2% lidocaine jelly (Xylocaine^®^ Jelly 2%, AstraZeneca, Södertälje, Sweden) prior to the procedure, followed by a subconjunctival injection of 1% lidocaine with 1:100,000 adrenaline (Xylocaine^®^ with Adrenaline, AstraZeneca, Södertälje, Sweden) at the time of the surgery. No sedatives, anxiolytics, or other pharmacologic agents were administered during the perioperative period in any of the study groups.

Monitoring: Physiological parameters—including the systolic and diastolic blood pressure (SBP and DBP), heart rate (HR), respiratory rate (RR), and oxygen saturation (SpO_2_)—were assessed at two time points: (1) immediately before headphone placement and (2) 15 min after the surgery’s completion while the participants were still wearing the assigned headphones. All measurements were performed using a Nihon Kohden Life Scope Monitor (PVM-400; Nihon Kohden Tomioka Corporation, Tomioka, Japan) by a team of four rotating perioperative nurses trained in standardized measurement protocols to ensure consistency and minimize inter-provider variability.

All auditory interventions were embedded within a structured, nurse-led perioperative care protocol to ensure fidelity, standardization, and feasibility. A single trained perioperative nurse was responsible for administering the audio intervention, including device setup, initial volume calibration, and comfort verification. Participant masking was preserved across all the groups through the use of visually identical headphones and standardized implementation procedures, as outlined in Section 2.1. The detailed structure and auditory components of the SBB and PM tracks are provided in Appendix A.

### 2.3. Assessment of Anxiety

The State–Trait Anxiety Inventory (STAI), a widely validated instrument for assessing anxiety in clinical populations, was employed to evaluate the anxiolytic effects of the SBB intervention. The STAI consists of two subscales: the Trait Anxiety Scale (STAI-T), which measures the general susceptibility to anxiety, and the State Anxiety Scale (STAI-S), which assesses transient, situational anxiety. Each subscale comprises 20 items, yielding scores ranging from 20 to 80, with higher scores indicating more severe anxiety. The Thai-adapted version of the STAI was administered under a research license obtained from Mind Garden, Inc., in full compliance with all licensing and copyright requirements [15,16]. A sample layout of the Thai-adapted STAI-S form is provided in Supplementary Figure S1, which illustrates item structure and response format.

As part of the preoperative workflow on the day of surgery, the participants first completed baseline assessments—including medical history, physical examination, audiologic and ophthalmologic assessments—followed by the administration of the STAI-T. The STAI-S was then administered approximately 10 min before entry into the operating room, immediately prior to the start of the assigned auditory intervention. Following the surgery, physiological parameters were recorded while the participants were still listening to the audio stimulus. The STAI-S was subsequently re-administered immediately after headphone removal.

All the postoperative assessments were conducted within 15 min after the completion of the surgery. This standardized timing was implemented to ensure consistency across participants and to minimize variability in anxiety measurement. It also served to reduce potential confounding effects related to prolonged auditory exposure or environmental influences outside the intervention window.

### 2.4. Outcomes

As outlined in Section 2.3, the primary outcome of this study was the change in the state anxiety levels, assessed using the pre- and postoperative scores on the State–Trait Anxiety Inventory—State (STAI-S). This outcome was selected to capture the immediate anxiolytic effect of the auditory intervention.

The secondary outcomes included changes in the physiological parameters—namely the systolic and diastolic blood pressure (SBP and DBP), heart rate (HR), respiratory rate (RR), and oxygen saturation (SpO_2_)—measured at the same time points. In addition, the operative duration, intraoperative complications, and adverse events were recorded to facilitate safety monitoring and provide contextual clinical insights.

All the primary and secondary outcomes were pre-specified in the study protocol and consistently applied throughout the study period. The planned structure for comparative analysis of these outcomes is illustrated in Table S1 (Supplementary Materials). No amendments to the outcome measures were made following trial initiation, ensuring protocol adherence and data integrity.

### 2.5. Randomization and Blinding

Randomization Method: The random allocation sequence was generated using block randomization with a fixed block size of six to ensure balanced group distribution throughout the recruitment phase. A computer-generated randomization list was utilized for the first 108 participants, and the final 3 were assigned to their groups using simple randomization with a 1:1:1 allocation ratio. All the allocations were performed by a single designated perioperative nurse responsible for managing the randomization process and intervention setup.

Allocation Concealment: The randomization list was securely stored in a password-protected database accessible only to a single designated perioperative nurse. The personnel involved in participant enrollment, the operating ophthalmologist, and the team of four rotating perioperative nurses did not have access to the allocation sequence. This approach ensured rigorous allocation concealment and minimized the risk of selection bias.

Implementation: The randomization sequence was prepared by the study statistician. Participants were enrolled by trained OPD nurses who were not involved in the intervention delivery or outcome assessment and remained blinded to the participants’ group allocation. Group allocation and delivery of auditory interventions were performed by the same designated perioperative nurse, who was structurally separated from the outcome assessment and surgical activities. This procedural separation was implemented to preserve methodological independence and reduce the potential for performance and detection bias.

Blinding: The participants, the operating ophthalmologist, and the perioperative nursing team—including the four rotating nurses responsible for physiological monitoring and questionnaire administration—were all blinded to the group allocation. The auditory interventions (SBBs, PM, and silence) were administered using visually identical audio devices and noise-canceling headphones to ensure both visual and auditory masking. Although the intervention nurse could not be blinded due to the nature of the task, her involvement was strictly confined to group assignment and audio setup. All the outcome assessments were performed by blinded personnel, and the data analyst remained unaware of the group allocations. The use of standardized protocols and indistinguishable equipment further minimized the potential for bias across the study arms.

### 2.6. Statistical Analysis

Sample size was calculated using the power command in Stata version 19.0 (StataCorp LLC., College Station, TX, USA), with a significance level (α) of 0.05 and statistical power set at 80% (β = 0.20). Reference values were obtained from a prior study by Opartpunyasarn et al. [17], which reported a mean reduction in the STAI-S score of 7.26 ± 9.31 in the intervention group and 1.12 ± 9.21 in the control group. Assuming equal group allocation (1:1 ratio), the minimum required sample size was determined to be 29 participants per group. To account for an anticipated 20% dropout rate, the final sample size was increased to 37 per group, resulting in a total of 111 participants across the three study arms. A one-sided hypothesis test was applied during power estimation.

All statistical analyses were conducted using Stata version 19.0 (StataCorp LLC., College Station, TX, USA). The categorical variables were compared between the groups using Fisher’s exact test, which is appropriate for small sample sizes or low expected cell counts. The overall group differences for continuous variables were assessed using a one-way analysis of variance (ANOVA). Linear regression models were also applied to evaluate the effect of group assignment on changes in state anxiety (STAI-S) scores and physiological outcomes, including SBP, DBP, HR, RR, and SpO_2_. Where significant group differences were observed, pairwise comparisons were performed using Bonferroni-adjusted post hoc tests. Within-group comparisons of the pre- and post-intervention outcomes were conducted using paired *t*-tests. A *p*-value of <0.05 was considered statistically significant for the global tests, while a stricter threshold of *p* < 0.0167 was applied for the pairwise comparisons to control for type I errors.

All the analyses followed the intention-to-treat (ITT) principle, whereby the participants were analyzed in the groups to which they were originally assigned, regardless of their intervention adherence. This approach preserved the benefits of randomization and enhanced the external validity of the results. No interim analyses were conducted, and no formal stopping rules were implemented. Missing data were handled using listwise deletion, and no imputation methods were applied.

## 3. Results

**Figure 1:**
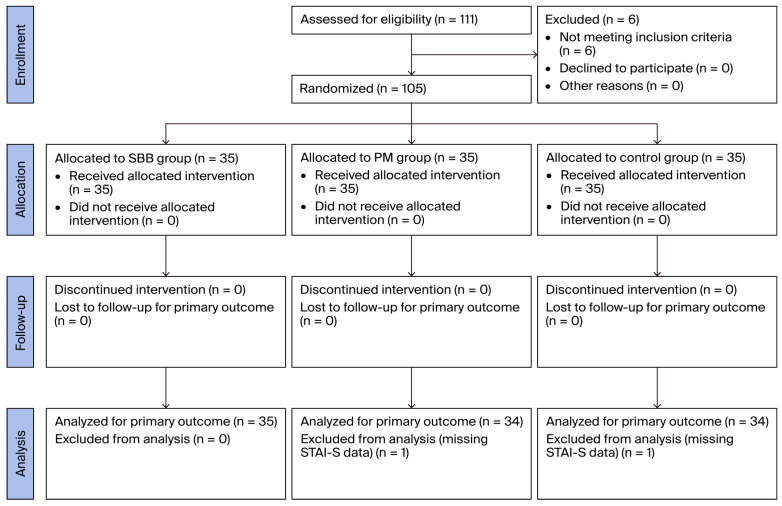
CONSORT flow diagram of study subjects.

All the participants were recruited between January and May 2025. A total of 111 patients scheduled for elective pterygium excision were screened for their eligibility. Six were excluded for not meeting the inclusion criteria—two due to documented auditory impairments and four due to a baseline blood pressure exceeding 160/100 mmHg. The remaining 105 eligible participants were randomly assigned to three groups using a computer-generated randomization sequence: the SBB group (*n* = 35), PM group (*n* = 35), and control group (*n* = 35). Two participants—one from the PM group and one from the control group—were excluded from the final analysis due to missing postoperative STAI-S data, resulting in the inclusion of 103 participants in the final analysis (Figure 1).

All the surgical procedures were performed by a single ophthalmologist to ensure consistency in the operative technique. A team of four rotating perioperative nurses implemented a standardized protocol encompassing preoperative assessments (of vital signs and the STAI-T/STAI-S), intraoperative nursing support, and postoperative evaluations (of vital signs and the STAI-S), all of which were integrated into routine clinical care.

The auditory interventions were administered according to standardized protocols by a single trained perioperative nurse, who also managed the group assignments. This approach ensured fidelity to the intervention protocol, minimized inter-provider variability, and standardized the headphone placement, audio setup, volume calibration, and timing across all the participants. All the participants completed their assigned auditory interventions without interruption, and no cases of premature headphone removal or protocol deviations were recorded.

The postoperative assessments—including the determination of STAI-S scores and measurement of physiological parameters—were completed within approximately 15 min of the surgery’s completion, although the 60 min auditory track had not necessarily concluded. The participants continued to wear the headphones during the postoperative assessments.

All participants received standard perioperative care, including the topical application of 2% lidocaine jelly and subconjunctival injection of 1% lidocaine with 1:100,000 adrenaline. No sedatives, anxiolytics, or additional pharmacologic agents were administered during the perioperative period in any group.

The trial proceeded as planned without interim analyses or early termination and concluded once the predefined sample size specified in the study protocol had been achieved.

The baseline psychological and physiological measures were comparable across the groups. The initial STAI-T scores showed no significant differences (Table 1), and the pre-intervention STAI-S scores were similar among the groups: 37.5 ± 5.5 in the SBB group, 36.5 ± 4.5 in the PM group, and 36.1 ± 5.2 in the control group (*p* = 0.525; Table 1). Likewise, the baseline physiological parameters—including the SBP (*p* = 0.945), DBP (*p* = 0.874), HR (*p* = 0.838), RR (*p* = 0.276), and SpO_2_ (*p* = 0.545)—did not differ significantly (Table 1).

Following the intervention, the SBB group demonstrated the greatest reduction in their STAI-S scores (−9.7 (95% CI −12.0 to −7.5)), followed by the PM group (−4.0 (95% CI −6.2 to −1.8)) and the control group (−0.9 (95% CI −2.4 to −0.7)), with a statistically significant overall group difference (*p* < 0.001; Table 2).

**Figure 2:**
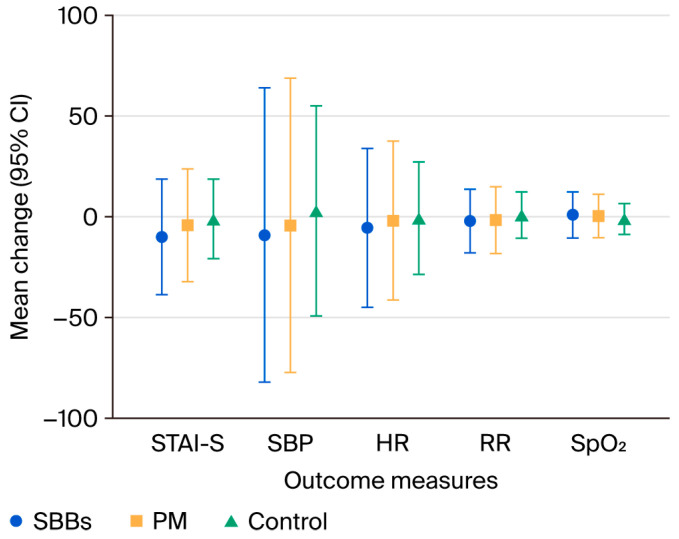
Mean change in outcome measures (State–Trait Anxiety Inventory—State (STAI-S), systolic blood pressure (SBP), heart rate (HR), respiratory rate (RR), and oxygen saturation (SpO_2_)) across three groups: superimposed binaural beats (SBBs), plain music (PM), and control (silence). Data are presented as mean differences from the baseline with 95% confidence intervals (CIs). Negative values indicate reductions from the baseline. The symbol notation is as follows: ● SBBs (*n* = 35); ■ PM (*n* = 35); ▲ control (*n* = 34). All outcome measures were assessed pre- and post-intervention. STAI-S = State–Trait Anxiety Inventory—State; SBP = systolic blood pressure; HR = heart rate; RR = respiratory rate; SpO_2_ = oxygen saturation. SBBs = superimposed binaural beats; PM = plain music.

In terms of physiological responses, the SBB group exhibited significant reductions in their systolic blood pressure (SBP; −8.9 (95% CI −14.6 to −3.1) mmHg, *p* = 0.011), heart rate (HR; −5.3 (95% CI −8.4 to −2.2) bpm, *p* = 0.003), and respiratory rate (RR; −1.9 (95% CI −3.2 to −0.7) breaths/min, *p* = 0.009). A modest increase in their oxygen saturation (SpO_2_; 1.2% (95% CI 0.3 to 2.1), *p* = 0.025) was also observed, whereas changes in their diastolic blood pressure (DBP; −3.1 (95% CI −6.6 to 0.4) mmHg, *p* = 0.222) were not statistically significant (Table 2; Figure 2).

The Bonferroni-adjusted post hoc analyses confirmed that the SBB group experienced significantly greater reductions in their STAI-S scores (*p* < 0.001), SBP (*p* = 0.008), HR (*p* = 0.003), and RR (*p* = 0.010) compared to the control group. However, the differences in their DBP (*p* = 0.250) and SpO_2_ (*p* = 0.020) did not meet the corrected significance threshold (*p* < 0.0167). No statistically significant pairwise differences were observed between the SBB and PM groups or between the PM and control groups for any of the measured outcomes (Table 3).

No adverse events, protocol violations, or unintended effects were reported during the intervention or surgical procedure in any group.

## 4. Discussion

This randomized controlled trial provided novel evidence supporting the effectiveness of a structured, nurse-led auditory intervention using SBBs to reduce perioperative anxiety in patients undergoing pterygium surgery under local anesthesia. The primary outcome, defined as the change in state anxiety levels measured by the State–Trait Anxiety Inventory—State (STAI-S), showed the most substantial reduction in the SBB group compared to both the PM and silence groups, thereby confirming the anxiolytic efficacy of the intervention.

Beyond the psychological effects, improvements in physiological parameters were also observed, suggesting a broader therapeutic potential of SBBs. These findings highlight the feasibility of integrating auditory-based, nurse-deliverable interventions into routine perioperative care—particularly in outpatient ophthalmic settings where pharmacologic anxiolytics may be impractical or unavailable.

To our knowledge, this is the first study to evaluate the clinical impact of a nurse-led SBB intervention in the context of pterygium excision. All participants underwent conjunctival autografting—a surgical technique requiring prolonged operative time and heightened patient cooperation—underscoring the importance of effective, non-pharmacological anxiety-reduction strategies in supporting procedural compliance during complex ophthalmic procedures.

### 4.1. Comparison with the Existing Literature

The present findings align with an expanding body of literature supporting the use of binaural beat (BB) stimulation as an effective, non-pharmacological strategy for perioperative anxiety reduction across various surgical settings. In obstetrics, Parodi et al. [18] demonstrated that exposure to BBs significantly alleviated anxiety in women undergoing elective cesarean section—an intervention often coordinated by nursing personnel. Similar anxiolytic effects have been observed in dental practice. Menziletoglu et al. [19] and Isik et al. [20] both reported meaningful reductions in preoperative dental anxiety following exposure to BB stimuli, highlighting the applicability of BB stimulation in short-duration, nurse-coordinated procedures.

In the field of urology, Ölçücü et al. [21] found that exposure to pure 10 Hz BBs significantly reduced both anxiety and pain in patients undergoing diagnostic cystoscopy and ureteral stent removal under local anesthesia. Likewise, Demirci et al. [22] reported that BBs prior to unsedated upper gastrointestinal endoscopy significantly reduced state anxiety and improved procedural tolerance, highlighting the applicability of BBs in minimally invasive, sedation-free procedures.

Taken together, these studies support the present findings and suggest that SBBs may offer enhanced anxiolytic effects over PM alone, particularly when implemented by nursing personnel trained in surgical care.

### 4.2. Nursing Implications and Feasibility

The integration of SBBs into perioperative nursing workflows offers a feasible, safe, and non-pharmacological approach to managing anxiety in outpatient surgical settings. In this study, trained perioperative nurses successfully administered the intervention—including device setup, auditory calibration, and monitoring—without protocol deviations or adverse events, supporting its practicality in routine clinical care.

The straightforward implementation—requiring only standard audio equipment, brief training, and minimal setup—may facilitate broader adoption in high-volume or resource-limited environments. Importantly, the patients remained alert and communicative throughout the procedure, maintaining intraoperative safety while benefiting from reduced anxiety. These characteristics align with the growing role of perioperative nurses in delivering sensory-based interventions that support emotional regulation without disrupting workflow.

Recent evidence supports this model. Zuo et al. [23] demonstrated that structured nursing interventions improved psychological outcomes and satisfaction in elderly cataract patients. Wu et al. [24] reported enhanced cooperation and efficiency in day-surgery settings following nurse-led perioperative management. Salazar Maya [25] emphasized the importance of non-verbal therapeutic presence in reducing anxiety during conscious procedures.

Building on this foundation, the delivery of SBBs may enhance emotional stability and improve communication between nurses and patients during awake surgeries. As a nurse-deliverable, non-invasive modality, SBBs can be integrated into perioperative care with minimal resource demands. Prior research across specialties—such as obstetrics, dentistry, urology and endoscopy—has shown that similar auditory interventions can reduce anxiety and improve patient cooperation [18,19,20,21,22].

Overall, these findings suggest that SBBs represent a viable addition to evidence-based nursing protocols for perioperative anxiety management. Their ease of use, compatibility with existing workflows, and positive patient reception underscore their potential value, particularly in outpatient or ambulatory surgical contexts.

### 4.3. Mechanism of Action and Physiological Relevance

The underlying neurophysiological mechanism of SBBs is believed to involve the frequency-following response (FFR), wherein cortical activity synchronizes with external auditory rhythms. The alpha frequency band (8–13 Hz), which was the target of this intervention, has been consistently associated with reduced arousal and increased relaxation [26]. To facilitate neural entrainment during the perioperative period, the auditory track was designed to shift gradually from a beta frequency (20 Hz) to an alpha frequency (10 Hz). This progressive transition mirrors the brain’s natural descent from alertness to calmness and may offer greater comfort and compliance than initiating with alpha frequencies alone.

Physiological improvements observed in the SBB group—including significant reductions in SBP, HR, and RR—suggest modulation of the autonomic nervous system (ANS) [27]. These changes are clinically relevant and readily trackable through standard perioperative monitoring, reinforcing the practicality of SBBs in everyday nursing practice.

Although not all pairwise comparisons between the SBB and PM groups reached statistical significance, the SBB group consistently demonstrated a greater magnitude of change across multiple parameters. This trend suggests a possible additive effect of binaural stimulation beyond that of music alone and supports the role of SBBs in enhancing neural entrainment.

A modest increase in SpO_2_ was also noted in the SBB group, although the difference did not remain statistically significant following Bonferroni correction. This pattern may reflect improved ventilatory efficiency or reduced sympathetic tone. Nonetheless, caution is advised in interpreting this finding, as SpO_2_ readings can be influenced by technical factors such as patient positioning, ambient temperature, and sensor placement [28].

Taken together, these findings suggest that structured SBB exposure may produce measurable physiological effects consistent with reduced sympathetic activation and enhanced relaxation—mechanisms that are both meaningful and readily observable within perioperative nursing contexts.

### 4.4. Study Strengths and Nursing Applicability

This study incorporates several strengths that enhance both the trustworthiness and clinical applicability of its findings. The use of a randomized controlled trial (RCT) design—with concealed allocation and blinded outcome assessment—minimized potential sources of bias and strengthened the internal validity of the results. Standardized protocols for surgical care, nursing intervention delivery, and auditory setup ensured consistency across all study conditions.

To maintain intervention fidelity, the SBB protocol was administered by a single trained nurse, while outcome assessments were conducted by personnel blinded to group assignments. These methodological safeguards helped ensure that the observed effects were attributable to the intervention itself rather than external or procedural factors.

Importantly, the intervention was well tolerated, with no adverse events or protocol deviations reported. This favorable safety profile supports the feasibility of implementing SBBs in broader clinical settings. Given their non-invasive nature, ease of administration, and minimal resource requirements, SBBs may be especially suitable for nurse-led environments—such as outpatient clinics, preoperative assessment units, and ambulatory surgical centers—where nurses play a central role in preparing and supporting patients.

Furthermore, the study achieved an exceptionally high follow-up rate, with nearly all participants completing both pre- and postoperative assessments. This outcome reflects the nature of the one-day surgical protocol—where data collection was integrated seamlessly into the standard perioperative workflow—and highlights the advantages of nurse-led coordination in ensuring participant retention and data completeness.

The structured design and encouraging outcomes of this study provide a foundation for integrating SBBs into evidence-informed nursing practice as a safe, scalable, and patient-centered strategy.

### 4.5. Study Limitations and Directions for Future Research

While the present study offers meaningful insights into the use of SBBs for perioperative anxiety reduction, several limitations should be acknowledged to guide future investigations. First, the study focused exclusively on anxiety, without assessing other psychological domains commonly affected in surgical contexts, such as stress, fear, or mood disturbances. Broadening the scope of psychological evaluation could provide a more comprehensive understanding of the intervention’s benefits.

Second, the subjective experiences of the participants were not explored. Although objective and validated measures demonstrated favorable outcomes, patient perspectives on comfort, acceptability, and perceived effectiveness remain unknown. Incorporating qualitative approaches—such as interviews or structured feedback—may enrich future research by capturing these experiential dimensions.

Third, while the intervention was designed to promote neurophysiological entrainment, no direct assessments such as electroencephalography (EEG) were utilized. Employing such tools in future studies would help validate the proposed mechanisms of action.

Moreover, the study population was limited to individuals undergoing pterygium excision under local anesthesia at a single outpatient site. To enhance generalizability, future research should explore the use of SBBs in a wider range of ambulatory procedures—such as cataract extraction, dental surgery, ENT operations, and minor dermatologic interventions—where patients are awake and perioperative anxiety is often pronounced.

The identical final group sizes (*n* = 35 per group) were an intended result of employing block randomization with a fixed block size, as specified in the study protocol. This method ensured equal distribution and minimized temporal imbalances across groups. We acknowledge that the perfect group balance could be perceived as allocation manipulation and have therefore addressed this issue for clarity.

Additionally, the study was conducted over a relatively short period (January–May 2025) due to the nature of the one-day surgical intervention, in which both the pre- and post-surgical assessments were completed within the same visit. While this compressed timeline enabled efficient data collection, it may be perceived as a limitation in terms of extended follow-up and broader validation.

Lastly, although the intervention was well tolerated, with no reported adverse events, the long-term effects and optimal delivery parameters—such as frequency range, session duration, and repetition schedules—remain to be clarified. Continued exploration of these factors will be essential in informing the safe, effective, and widely applicable integration of SBBs into routine perioperative care.

## 5. Conclusions

This study provides evidence that superimposed binaural beats (SBBs), delivered through a structured, nurse-led auditory intervention, may effectively reduce perioperative anxiety and support physiological stability in patients undergoing pterygium surgery under local anesthesia. Participants in the SBB group demonstrated greater improvements in both subjective anxiety scores (STAI-S) and objective physiological parameters (SBP, HR, and RR) compared to those in the plain music and silence groups, suggesting an enhanced effect beyond conventional music therapy.

As a safe, non-invasive, and low-cost modality, SBBs can be readily integrated into perioperative workflows without the need for additional pharmacologic agents. Their successful implementation by trained perioperative nurses underscores the expanding role of nursing professionals in delivering evidence-based, patient-centered interventions that enhance emotional readiness and promote procedural cooperation.

Future research should aim to validate the neurophysiological mechanisms underlying SBBs—particularly through electroencephalographic (EEG) assessments—to confirm brainwave entrainment. Comparative studies with pharmacologic anxiolytics and longitudinal evaluations of patient-reported outcomes are also warranted. Expanding the scope of nurse-led sensory interventions like SBBs may foster more responsive, scalable, and sustainable models of care in outpatient surgical settings.

## Figures and Tables

**Table 1:** Demographic data.

Variable	SBBs (*n* = 35)	PM (*n* = 34)	Control (*n* = 34)	*p*-Value
Age (years)	57.5 ± 9.6	57.5 ± 9.3	57.8 ± 10.0	0.991
Sex				
Male	7 (20.0%)	9 (26.5%)	11 (32.4%)	0.500
Underlying disease				
DM	6 (17.1%)	4 (11.8%)	7 (20.6%)	0.640
HT	11 (31.4%)	12 (35.3%)	9 (26.5%)	0.747
CVD	0 (0.0%)	1 (2.9%)	2 (5.9%)	0.320
Others	7 (20.0%)	12 (35.3%)	8 (23.5%)	0.335
Drug use				
Beta blocker	2 (5.7%)	0 (0.0%)	2 (5.9%)	0.542
Anxiolytic drug	0 (0.0%)	1 (2.9%)	1 (2.9%)	0.547
BMI (kg/m^2^)	25.3 ± 4.8	25.1 ± 4.3	23.6 ± 3.5	0.192
ASA classification				
I	15 (42.9%)	15 (44.1%)	17 (50.0%)	0.880
II	20 (57.1%)	19 (55.9%)	17 (50.0%)	
Pterygium grading				
II	21 (60.0%)	23 (67.7%)	20 (58.8%)	0.885
III	7 (20.0%)	7 (20.6%)	7 (20.6%)	
IV	7 (20.0%)	4 (11.8%)	7 (20.6%)	
STAI-T scores	42.0 ± 3.8	42.8 ± 4.0	42.5 ± 4.9	0.764
Operation time (minutes)	25.2 ± 4.5	25.7 ± 5.3	25.0 ± 4.9	0.808

Values are presented as mean ± SD and number (%). SBBs = superimposed binaural beats; PM = plain music; DM = diabetes mellitus; HT = hypertension; CVD = cardiovascular disease; BMI = body mass index; ASA = American Society of Anesthesiologists; STAI-T = State–Trait Anxiety Inventory—Trait.

**Table 2:** STAI-S scores, SBP, DBP, HR, RR, and SpO_2_.

Variable	SBBs (*n* = 35)	PM (*n* = 35)	Control (*n* = 34)	*p*-Value
STAI-S scores				
Pre-intervention	37.5 ± 5.5	36.5 ± 4.5	36.1 ± 5.2	0.525
Post-intervention	26.9 ± 3.7	31.6 ± 4.5	35.3 ± 4.9	<0.001
Mean change (95% CI)	−9.7 (−12.0, −7.5)	−4.0 (−6.2, −1.8)	−0.9 (−2.4, 0.7)	<0.001
*p*-value (within-group)	<0.001	<0.001	0.130	
SBP				
Pre-intervention	139.5 ± 16.2	140.8 ± 16.0	140.4 ± 16.0	0.945
Post-intervention	133.9 ± 16.0	139.9 ± 15.5	143.6 ± 18.3	0.054
Mean change (95% CI)	−8.9 (−14.6, −3.1)	−4.1 (−9.8, 1.7)	3.2 (−0.9, 7.3)	0.011
*p*-value (within-group)	0.005	0.336	0.934	
DBP				
Pre-intervention	82.1 ± 9.1	83.2 ± 9.4	83.1 ± 10.6	0.874
Post-intervention	80.8 ± 10.3	83.4 ± 9.5	84.9 ± 9.9	0.224
Mean change (95% CI)	−3.1 (−6.6, 0.4)	−1.6 (−5.2, 1.9)	1.8 (−0.7, 4.3)	0.222
*p*-value (within-group)	0.157	0.556	0.910	
HR				
Pre-intervention	71.3 ± 12.0	72.0 ± 10.8	73.0 ± 12.6	0.838
Post-intervention	65.5 ± 9.9	69.8 ± 10.0	72.5 ± 11.4	0.026
Mean change (95% CI)	−5.3 (−8.4, −2.2)	−1.7 (−4.8, 1.4)	−0.5 (−2.7, 1.7)	0.003
*p*-value (within-group)	<0.001	0.022	0.313	
RR				
Pre-intervention	17.1 ± 3.6	15.8 ± 3.5	17.1 ± 4.1	0.276
Post-intervention	16.2 ± 2.9	15.4 ± 3.3	18.1 ± 3.6	0.003
Mean change (95% CI)	−1.9 (−3.2, −0.7)	−1.5 (−2.8, −0.2)	1.1 (0.2, 2.0)	0.009
*p*-value (within-group)	0.035	0.222	0.997	
SpO_2_				
Pre-intervention	97.4 ± 1.8	97.8 ± 1.5	97.4 ± 1.7	0.545
Post-intervention	97.8 ± 1.9	97.6 ± 1.3	96.6 ± 1.8	0.009
Mean change (95% CI)	1.2 (0.3, 2.1)	0.6 (−0.2, 1.5)	−0.8 (−1.4, −0.2)	0.025
*p*-value (within-group)	0.846	0.253	0.002	

Values are presented as mean ± SD and mean change (95% CI). SBBs = superimposed binaural beats; PM = plain music; STAI-S = State–Trait Anxiety Inventory—State; SBP = systolic blood pressure; DBP = diastolic blood pressure; HR = heart rate; RR = respiratory rate; SpO_2_ = oxygen saturation.

**Table 3:** Comparison of mean changes in STAI-S scores, SBP, DBP, HR, RR, and SpO_2_ across study groups.

Variable	Mean Change (95% CI)			*p*-Value		
	SBBs	PM	Control	SBBs vs. PM	SBBs vs. Control	PM vs. Control
STAI-S scores	−9.7 (−12.0, −7.5)	−4.0 (−6.2, −1.8)	−0.9 (−2.4, 0.7)	<0.001	<0.001	0.002
SBP	−8.9 (−14.6, −3.1)	−4.1 (−9.8, 1.7)	3.2 (−0.9, 7.3)	0.295	0.008	0.494
DBP	−3.1 (−6.6, 0.4)	−1.6 (−5.2, 1.9)	1.8 (−0.7, 4.3)	1.000	0.250	1.000
HR	−5.3 (−8.4, −2.2)	−1.7 (−4.8, 1.4)	−0.5 (−2.7, 1.7)	0.067	0.003	0.866
RR	−1.9 (−3.2, −0.7)	−1.5 (−2.8, −0.2)	1.1 (0.2, 2.0)	1.000	0.010	0.070
SpO_2_	1.2 (0.3, 2.1)	0.6 (−0.2, 1.5)	−0.8 (−1.4, −0.2)	0.588	0.020	0.450

Values are presented as mean change (95% CI). SBBs = superimposed binaural beats; PM = plain music; STAI-S = State–Trait Anxiety Inventory—State; SBP = systolic blood pressure; DBP = diastolic blood pressure; HR = heart rate; RR = respiratory rate; SpO_2_ = oxygen saturation.

## Data Availability

The data are available from the corresponding author upon reasonable request.

## Supplementary Materials

The following supporting information can be downloaded at https://www.mdpi.com/article/10.3390/nursrep15080282/s1: Figure S1. Sample format of Thai-adapted State–Trait Anxiety Inventory—State (STAI-S), showing item layout and response scale. Note: Only a partial view is presented for illustrative purposes due to copyright restrictions. The full version is available upon request with appropriate permissions. Table S1: Dummy table illustrating planned structure for comparative analysis of primary (STAI-S scores) and secondary (physiological) outcomes. This table does not include actual patient data.

## Public Involvement Statement

No patients or members of the public were involved in any aspect of this research.

## Guidelines and Standards Statement

This manuscript was drafted in accordance with the CONSORT 2025 Statement for reporting parallel-group randomized trials.

## Abbreviations

The following abbreviations are used in this manuscript:

ANOVA	Analysis of Variance
ANS	Autonomic Nervous System
ASA	American Society of Anesthesiologists
BBs	Binaural Beats
BMI	Body Mass Index
DBP	Diastolic Blood Pressure
EEG	Electroencephalography
ENT	Ear, Nose, and Throat
FFR	Frequency-Following Response
HR	Heart Rate
ITT	Intention to Treat
OPD	Outpatient Department
PM	Plain Music
RCT	Randomized Controlled Trial
RR	Respiratory Rate
SBBs	Superimposed Binaural Beats
SBP	Systolic Blood Pressure
SpO_2_	Oxygen Saturation
STAI-S	State–Trait Anxiety Inventory—State
STAI-T	State–Trait Anxiety Inventory—Trait
TCTR	Thai Clinical Trials Registry

## Appendix A

### Appendix A.1. Superimposed Binaural Beat (SBB) Audio Composition

The SBB track used in this study followed the structure below:

- Initiation at 20 Hz (beta range) for alertness reduction: 0–5 min
- A gradual shift to 10 Hz (alpha range): 5–10 min
- Maintenance at 10 Hz: 10–60 min
- Continuation for 15 min: postoperative.

The audio was generated using the SHARM self-hypnosis system (Cyber Team Ltd.), combining ambient natural sounds (e.g., forest and ocean sounds) with sine-wave-based binaural frequency differentials. The SBB track was custom-developed and licensed for academic research use by Chiang Mai University, Thailand. Redistribution or reproduction outside the scope of this study is not permitted.

### Appendix A.2. Plain Music (PM) Audio Composition

The plain music (PM) track used in this study was identical to the SBB version in terms of the musical composition, ambient nature sounds, audio duration, and volume. However, all binaural frequency differentials were omitted. The PM track retained the same structural flow and auditory environment as the SBB version to ensure consistency across the intervention groups while isolating the effect of binaural beat stimulation. This audio was also developed and licensed for research purposes by Chiang Mai University.
