# Supplementary material for: Nurse-Led Binaural Beat Intervention for Anxiety Reduction in Pterygium Surgery: A Randomized Controlled Trial
Source: Nurs Rep. 2025 Jul 31;15(8):282. doi: 10.3390/nursrep15080282 (PMC12389307; doi:10.3390/nursrep15080282)
Supplement: Supplementary file 1 [file nursrep-15-00282-s001.zip › Figure S1_Supplementary_Materials.pdf]

Figure S1.

Sample layout of the Thai-adapted State–Trait Anxiety Inventory-State (STAI-S), presenting item structure and bilingual response scale.

Note: This is a partial view provided for illustrative purposes. The full version is available upon request with appropriate permission.

**ข้อที่**<br>**Item No.**	**รายการคำถาม (ตัวอย่าง)**<br>**Sample Item (Thai)**	**ไม่เลย (1)**<br>**Not at all**	**เล็กน้อย (2)**<br>**Somewhat**	**ปานกลาง (3)**<br>**Moderately**	**มาก (4)**<br>**Very much**
1	ฉันรู้สึก<br>I feel calm				
2	ฉันรู้สึกมั่นคง<br>I feel secure				
3	ฉันรู้สึกตึงเครียด<br>I feel tense				
