# Supplementary material for: Nurse-Led Binaural Beat Intervention for Anxiety Reduction in Pterygium Surgery: A Randomized Controlled Trial
Source: Nurs Rep. 2025 Jul 31;15(8):282. doi: 10.3390/nursrep15080282 (PMC12389307; doi:10.3390/nursrep15080282)
Supplement: Supplementary file 1 [file nursrep-15-00282-s001.zip › Table S1_Supplementary_Materials.pdf]

Table S1.

Planned structure for comparative analysis of primary (STAI-S scores) and secondary (physiological) outcomes. This dummy table does not contain actual patient data.

ตารางตัวอย่างนี้แสดงโครงสร้างของการวิเคราะห์เปรียบเทียบของผลลัพธ์หลัก (คะแนน STAI-S) และผลลัพธ์รอง (ค่าทางสรีรวิทยา) โดยไม่มีการใช้ข้อมูลจากผู้ป่วยจริง

**กลุ่ม Group**	**จำนวน (*n*)**	**คะแนน STAI-S ก่อนผ่าตัด Pre-op STAI-S**	**คะแนน STAI-S หลังผ่าตัด Post-op STAI-S**	**ผลต่าง Mean Change in STAI-S**	**ผลต่าง Mean Change in SBP (มม.ปรอท / mmHg)**	**ผลต่าง Mean Change in DBP (มม.ปรอท / mmHg)**	**ผลต่าง Mean Change in HR (ครั้ง/นาที / bpm)**	**ผลต่าง Mean Change in RR (ครั้ง/นาที / breaths/min)**	**ผลต่าง Mean Change in SpO_2 (%)**
SBBs									
Plain Music									
Control									
